# Perception of midline deviations in smile esthetics by laypersons

**DOI:** 10.1590/2177-6709.21.6.051-057.oar

**Published:** 2016

**Authors:** Jamille Barros Ferreira, Licínio Esmeraldo da Silva, Márcia Tereza de Oliveira Caetano, Andrea Fonseca Jardim da Motta, Adriana de Alcantara Cury-Saramago, José Nelson Mucha

**Affiliations:** 1 Master’s degree in Orthodontics, Universidade Federal Fluminense, Niterói/RJ, Brazil.; 2 Professor, Statistics Department, Universidade Federal Fluminense, Niterói/RJ, Brazil.; 3 Professor, Dental Clinics Department, Universidade Federal Fluminense, Niterói/RJ, Brazil.; 4 Full professor, Dental Clinics Department, Universidade Federal Fluminense, Niterói/RJ, Brazil.

**Keywords:** Esthetics, dental, Photography, dental, Perception, Orthodontics

## Abstract

**Objective:**

To evaluate the esthetic perception of upper dental midline deviation by laypersons and if adjacent structures influence their judgment.

**Methods:**

An album with 12 randomly distributed frontal view photographs of the smile of a woman with the midline digitally deviated was evaluated by 95 laypersons. The frontal view smiling photograph was modified to create from 1 mm to 5 mm deviations in the upper midline to the left side. The photographs were cropped in two different manners and divided into two groups of six photographs each: group LCN included the lips, chin, and two-thirds of the nose, and group L included the lips only. The laypersons performed the rate of each smile using a visual analog scale (VAS). Wilcoxon test, Student’s t-test and Mann-Whitney test were applied, adopting a 5% level of significance.

**Results:**

Laypersons were able to perceive midline deviations starting at 1 mm. Statistically significant results (*p*< 0.05) were found for all multiple comparisons of the values in photographs of group LCN and for almost all comparisons in photographs of group L. Comparisons between the photographs of groups LCN and L showed statistically significant values (*p*< 0.05) when the deviation was 1 mm.

**Conclusions:**

Laypersons were able to perceive the upper dental midline deviations of 1 mm, and above when the adjacent structures of the smiles were included. Deviations of 2 mm and above when the lips only were included. The visualization of structures adjacent to the smile demonstrated influence on the perception of midline deviation.

## RESUMO

**Objetivo:**

este estudo teve por objetivo avaliar a percepção estética dos desvios da linha média dentária superior por indivíduos leigos, assim como se as estruturas adjacentes influenciam nesse julgamento.

**Métodos:**

um álbum contendo 12 fotografias frontais do sorriso de uma mulher, aleatoriamente distribuídas e com a linha média desviada digitalmente, foi avaliado por 95 indivíduos leigos. As fotografias frontais de sorriso foram modificadas para criar desvios de 1 mm a 5 mm para a esquerda na linha média superior. As fotografias foram obtidas de duas maneiras e divididas em dois grupos de seis fotografias cada: Grupo LCN, incluindo lábios, mento e dois terços do nariz; e Grupo L, incluindo apenas os lábios. Os indivíduos leigos classificaram o sorriso de acordo com uma Escala Visual Analógica (EVA). Os testes de Wilcoxon, *t*de Student e Mann-Whitney foram aplicados, adotando-se um nível de significância de 5%.

**Resultados:**

os leigos foram capazes de perceber desvios da linha média a partir de 1 mm. Resultados estatisticamente significativos (*p* < 0,05) foram encontrados para todas as múltiplas comparações dos valores nas fotografias do Grupo LCN e para quase todas as comparações nas fotografias do Grupo L. As comparações entre as fotografias dos grupos LCN e L mostraram valores estatisticamente significativos (*p* < 0,05) quando o desvio foi de 1 mm.

**Conclusões:**

os indivíduos leigos foram capazes de perceber os desvios da linha média de 1 mm; e acima desses, quando estruturas adjacentes ao sorriso apareciam nas fotografias. Desvios de 2 mm ou mais foram percebidos apenas quando os lábios apareciam nas fotografias; a visualização das estruturas adjacentes ao sorriso pareceu ter influência sobre a percepção dos desvios da linha média.

## INTRODUCTION

The dental literature available on the esthetics of the face and smile is very wide and always been discussed among dental professionals as well as has become interesting to people of different cultures, social classes and ages.^1^ ^-^ ^5^ This interest is justified by the fact that persons with esthetically attractive smile have higher chances of acceptance by society, ensuring better interpersonal relations because they are considered friendly, popular, sociable and intelligent.^1^ ^,^ ^3^ ^,^ ^6^ ^-^ ^8^

However, esthetic perception of dental professionals do not always match the opinion of the patients and this different view implies that more research involving laypersons would help to better understand the perception and the esthetic effects of certain smile characteristics.^9^ ^-^ ^11^

Moreover, the importance of midline asymmetries on orthodontic diagnosis and treatment planning, is justified by the large number of cases with this malocclusion treated by orthodontists. Therefore, many studies have been done on the diagnosis and treatment of facial and dental asymmetry.^1^ ^,^ ^2^ ^,^ ^11^ ^-^ ^14^ An individual’s facial midline was defined by the soft tissue symmetry - base of the nose, nasal apex, center of the philtrum and central point of the chin^15^ -, and the upper dental midline is evaluated by locating the tip of the gingival papilla between the maxillary central incisors. The gingival papilla should be located below the center of the philtrum of the upper lip.^16^

Although a subtle asymmetry between the facial and dental midlines may exist within acceptable limits, significant discrepancies can alter the level of dental attractiveness and may be detrimental to facial esthetics.^17^ However, standards for evaluating midline discrepancy are difficult to established given the subjective nature of such assessment.^17^ ^,^ ^18^

Results from many studies that tried to determine the acceptability deviation of dental midline by dentists, orthodontists, patients, and laypersons are still conflicting.^17^ ^-^ ^19^ Some studies found that the laypersons had considered the midline deviations as acceptable only under 2 mm deviation,^18^ ^,^ ^20^ ^-^ ^22^ meanwhile other researches had found values around 3 mm to acceptability threshold.^15^ ^,^ ^22^ ^-^ ^24^ Other controversial studies have found that 4 mm or less in midline deviations could not be perceived by layperson.^12^ ^,^ ^15^ ^,^ ^25^

A few studies used digitally modified images to determine the laypersons perception of the details that influence on the attractiveness of the smile. Disagreements between the values for acceptability may be related to differences in images manipulation among studies, the presence or not of anatomical structures surrounding to smile, the chosen model for handling as well as the size of images.^18^ ^,^ ^22^ ^,^ ^24^ ^-^ ^26^

Different methodologies were applied to evaluate the esthetic perception of the midline deviation, such as the kind of evaluators selection,^12^ ^,^ ^15^ ^,^ ^22^ ^,^ ^25^ sample size,^4^ ^,^ ^12^ ^,^ ^15^ ^,^ ^18^ ^,^ ^20^ ^-^ ^23^ ^,^ ^25^ ^,^ ^27^ evaluators calibrated or not,^12^ ^,^ ^15^ ^,^ ^18^ ^,^ ^20^ ^-^ ^25^ different times for judgment,^12^ ^,^ ^15^ ^,^ ^20^ ^,^ ^21^ ^,^ ^23^ ^,^ ^24^ number of smiling subjects to be evaluated,^4^ ^,^ ^15^ ^,^ ^18^ ^,^ ^22^ ^,^ ^24^ ^,^ ^25^ photographs displayed size,^4^ ^,^ ^12^ ^,^ ^18^ ^,^ ^20^ ^-^ ^24^ ^,^ ^27^ with and without anatomical structures adjacent to the smile,^4^ ^,^ ^12^ ^,^ ^15^ ^,^ ^18^ ^,^ ^20^ ^-^ ^25^ ^,^ ^27^ amount of deviation in each studies,^12^ ^,^ ^18^ ^,^ ^20^ ^-^ ^25^ ^,^ ^27^ different ways to define what would be assessed: perception, attractiveness, more or less esthetic, among other expressions.^4^ ^,^ ^12^ ^,^ ^15^ ^,^ ^18^ ^,^ ^20^ ^-^ ^25^ ^,^ ^27^

The acceptable deviation determination in midline is essential for decision making by the orthodontist. The solution for existing deviations from the midline may involve tooth movement, with or without dental extractions, orthopedic treatment or the need for orthognathic surgery. In some cases, the correction of the dental and facial midline is not simple and may increase the complexity and duration of orthodontic treatment.^2^ ^,^ ^4^ ^,^ ^20^ Differential diagnosis makes it possible to discern the cause of the problem, enabling the use of proper mechanotherapy.^28^

Regardless of the orthodontists’ desire to achieve all the orthodontic treatment goals, is their commitment to get the patient satisfaction, and the esthetic factor is prioritized by patients in orthodontic treatment.^8^

Based on this premise, we proposed in this research to evaluate the esthetic perception of the upper dental midline deviation by a group of laypersons, and to determine the influence of viewing the structures adjacent to the smile, such as lips, chin and nose, on the diagnosis of the midline deviation.

## MATERIAL AND METHODS

This comparative and observational cross-sectional study was approved by the Ethics in Research Committee of the School of Medicine, *Universidade Federal Fluminense*, Niterói, Rio de Janeiro, Brazil, under control number 422.820.

One female subject with normal occlusion was selected among the residents at the postgraduate orthodontic residency program at *Universidade Federal Fluminense*, Niterói, Rio de Janeiro (Brazil), and agreed to participate in the study. 

The frontal smiling photograph of the subject was obtained with a digital camera (EOS 60D; Canon, Tokyo, Japan). The photograph was altered using Adobe Photoshop software (Adobe Systems Inc, San Jose, USA) and progressive changes were applied to the upper dental midline relative to the facial midline at every 1 mm, from 0 to 5 mm. By altering the dental midline the entire adjacent tissue was held in position while the whole upper arch was gradually shifted only to the left. 

The photographs were cropped in two different configurations and divided into two groups: Group LCN, including the lips, chin, and two thirds of the nose, and Group L, including the lips only. This resulted in twelve photographs for evaluation, two without midline shift and ten digitally altered that were standardized to replicate the subject’s smile in its original size (real scale).

**Figure 1:**
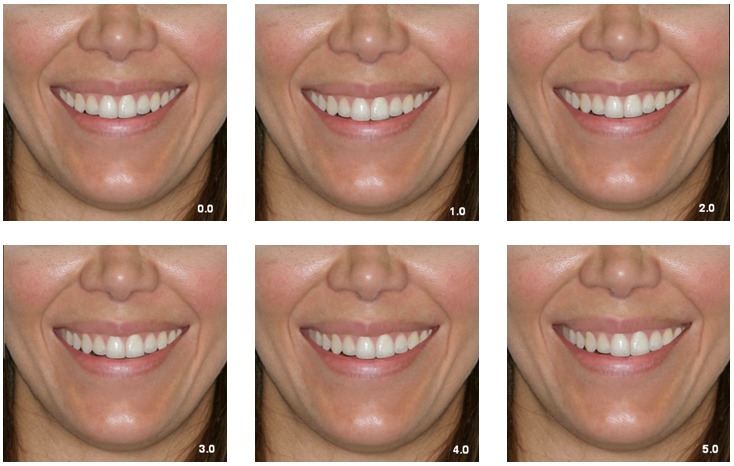
Group LCN photographs: the numbers on the photographs indicate the amount of deviation in millimeters

**Figure 2:**
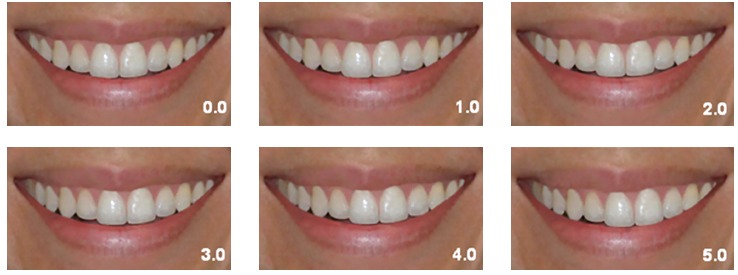
Group L photographs: the numbers on the photographs indicate the amount of deviation in millimeters.

The twelve digital photographs (six from group LCN and six from group L) were printed and randomly arranged in an album. The photographs were coded to avoid identification discrepancies. The first part of the album contained group LCN photographs, and the second part, group L (Figs 1 and 2). Photograph evaluation was performed by 95 laypersons with a mean age of 21 years and 3 months. The type of sampling was based on cluster randomization and the evaluators were directly recruited by the researcher in the order they get in the university campus, and they had complete freedom to participate or not of the research. None had undergone orthodontic treatment prior to starting the evaluations and had no experience in Dentistry. 

Before evaluating the photographs, a calibration was performed with the judges using two photographs, one without midline deviation (original) and one having a deviation of 6 mm to the left side. Visual Analog Scale (VAS) numbered from zero to 100 was used to mark the scores assigned to the photographs, with the lowest value assigned to the least esthetic smile and the highest value to the most esthetic. The mean value of 50 mm on the VAS was considered the cutoff between attractive and unattractive smile. The time limit for observing each photograph was 20 seconds with a maximum interval of ten seconds between photographs in order to enable the evaluators to assign a score to the smile on the VAS. The evaluators were instructed not to turn back to the previous page of the album to see a particular image again. 

After marking the values ​​assigned to the esthetics of the smile on their respective scales, measurements were performed by an operator with the aid of a digital caliper (Starret Indústria e Comércio Ltda., Itu, São Paulo) properly calibrated to the VAS, positioned at zero point, and extended as far as the marking made by the evaluator. 

A sample size calculation was performed using the formula recommended by Pandis,^29^ based on statistical power of 90% with a confidence interval of 95% (α = 0.05) and standard deviation (SD = 20.88 mm) described by Motta^14^ to detect a mean difference of 10 in VAS scores, which resulted in 92 evaluators. 

To verify the method error, 21 evaluators randomly selected (representing 22% of the total) were asked to repeat the assessment after a 2-weeks interval. Student’s *t*-test for paired samples was used for intrarater systematic error analysis, while intraclass correlation coefficient was applied to determine the calibration of the laypersons for photographs evaluation. 

The data were tabulated and analyzed using the Statistical Package for the Social Sciences^©^ software (SPSS Inc. Chicago, USA). Data normality was evaluated by the Kolmogorov-Smirnov statistical test. 

To assess the influence of changes in the upper dental midline on the perception of smile esthetics, the Friedman test, followed by the Wilcoxon test considering the level of significance as corrected by the Bonferroni criterion (α = 0.0033) were applied for multiple comparisons. Paired Student’s *t*-test was used whenever data were considered normal, and Friedman test when the data were not considered normal, followed by Mann-Whitney test to assess the impact of structures adjacent to the smile on the perception of deviation in the upper dental midline. The level of significance adopted was 5% (*p*< 0.05).

Pearson’s correlation coefficient and regression equation were formulated to determine the association between deviations in groups LCN and L, and the mean values ​​assigned by the evaluators. The coefficient of determination was calculated to predict the accuracy of the regression equation.

## RESULTS

The paired Student *t*-test used to evaluate the systematic error, showed no significant difference (*p*> 0.05) and the ICC (0.953) showed an excellent calibration of the laypersons who performed the photographs evaluations.

Statistically significant values were found for all multiple comparisons of the attractiveness scores assigned to each midline shift in photographs of group LCN (Table 1). In group L there were statistical significant differences for almost all comparisons (Table 1). The only exceptions occurred in group L when the photograph that had no deviation was compared with the photograph with a 1 mm shift, and between photographs with 2 mm and 3 mm shifts. 

**Table 1:** Descriptive statistics (mm) and results for attractiveness scores and for comparisons between LCN group (including the lips, chin and nose) and L group (including the lips only).

Deviation	LCN group				L group				LCN x L		
(mm)	Median	Mean	IQ	SD	Results*	Median	Mean	IQ	SD	Results*	
0	82.70	-	24.34	-	A	84.60	-	20.36	-	A	
1	75.66	-	22.51	-	B	80.26	-	18.55	-	A	†
2	70.31	-	23.91	-	C	70.54	-	28.43	-	B	
3	63.42	-	27.82	-	D	66.21	-	33.12	-	B	
4	51.68	-	35.28	-	E	53.68	-	36.93	-	C	
5	-	42.13	-	25.19	F	-	44.15	-	23.23	D	

*Variables with the same letter does not differ statistically (*p* < 0.05); † Statistical differences between groups of facial structures (*p* < 0.05).

**Figure 3:**
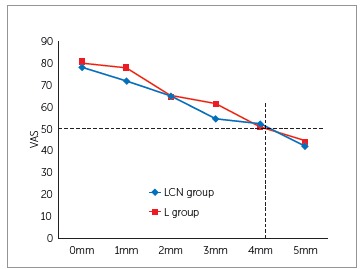
Comparison between the overall scores assigned to the photographs in LCN and L groups.

Results of the tests performed to verify the impact of structures adjacent to the smile on the perception of upper dental midline deviations showed statistically significant difference (*p*< 0.05) for comparisons between the photographs of groups LCN and L only when the deviation was 1 mm (Table 1, Fig 3). For other situations of the midline deviation, the mean scores did not differ (*p*> 0.05). 

The result of the Pearson’s correlation coefficient showed strong negative correlation among deviations in groups LCN and L, and the mean values ​​assigned by the evaluators (r = - 0.9963). The value of the coefficient of determination (r^2^ = 0.9926) and the linear regression equation (y = -7.366x + 80.741) were derived from the data collected for this study.

## DISCUSSION

Although common sense tends to base the concept of facial esthetics on subjective opinions, the qualitative and quantitative processing of scientific orthodontic data regarding what is considered beautiful and pleasing is an element that can improve communication with the patient in order to meet their expectations. As the concept of beauty is personal, hence subjective, it requires a fast, straightforward and reliable evaluation method. Therefore, a VAS was used as research tool by the evaluators in this study.^12^ ^-^ ^15^ ^,^ ^20^ ^,^ ^25^

The methodology employed in the present study used photographs with alterations in the upper dental midline only to the left.^14^ However, some authors who set out to evaluate the perception of the upper dental midline deviation also included the investigation of other potentially significant discrepancies in the smile attractiveness.^8^ ^,^ ^12^ ^,^ ^19^ ^,^ ^22^ ^-^ ^25^ ^,^ ^27^ This methodology may produce questionable results given that the inclusion of numerous distinct features could confuse the evaluator.

Facial features, such as hair color, face pattern, skin color and gender, are factors that potentially affect the level of visual attention on the smile esthetic perception by laypersons.^5^ ^,^ ^25^ Therefore, to gauge the interference of these structures of the face and evaluate the influence of structures that define the facial midline, two settings were applied to the photographs used in this study, which were divided into groups LCN and L. However, full face photographs were not employed.

The fact that they have assessed the photographs randomly and separately probably decreases the incorporation of bias. The evaluators could not compare the photographs at the same time like in previous studies,^22^ ^,^ ^23^ ^,^ ^26^ which might have contributed to the results found in this study, since the variation from the least esthetic value to the most esthetic were limited between 42.13 mm and 84.60 mm (Table 1).

According to the findings of our study, laypersons were more critical in the perception of changes of the upper dental midline in the photographs of LCN group. There were statistically significant differences for all multiple comparisons between each midline shift in photographs of LCN group. These results evidence the capacity of laypersons to perceive each millimeter of deviation in photographs of LCN group. However, there were statistically significant differences for some multiple comparisons between each midline shift on photographs of L group. These results show the perception of laypersons to note midline deviations only from 2 mm, when anatomical details are suppressed in photographs arranged for evaluation. Likewise the evaluators failed to differentiate shifts between 2 and 3 mm or may not have detected significant difference between these midline variations (Table 1).

This result probably stemmed from the fact that LCN group photographs contained anatomical landmarks of the face such as the lips, chin, and nose, which are natural contributors to the diagnosis of upper dental midline deviation. Some investigations, using photographs of the whole face for evaluation of upper dental midline deviation, found that laypersons were able to notice deviations starting at 2 mm.^4^ ^,^ ^18^ ^,^ ^20^ ^,^ ^21^ This divergence possibly resulted from the influence of other facial structures, which might potentially disperse the evaluation of smile esthetics by laypersons.^4^ ^,^ ^24^

Other studies analyzed the perception of dental midline deviations by laypersons in photographs showing only the smile, but with different methodologies. In the works of Ker et al^23^ and Mc Leod et al^22^, the evaluators accepted deviations in the upper midline of up to 2.9 mm, but they had judged all the photographs at the same time. Nevertheless, some studies reported that laypersons could only identify deviations from the upper midline of up to 3 mm^15^ ^,^ ^17^ and 4 mm.^15^ Furthermore, studies conducted by Kokich et al,^25^ with pictures showing just the smile, concluded that 4 mm deviations might not be detectable by laypersons. These divergent results may have been due to the different methodologies used in the investigations as well as the heterogeneity of the population being studied. 

In spite of the results of our study showing that the laypersons were able to identify deviations from the midline starting at 1 mm in LCN group and 2 mm in L group, it seems that only from a deviation of approximately 4 mm that the smile was considered not esthetically pleasing by laypersons. This can be explained by applying the mean value of 50 mm in the linear regression equation (y = -7.366x + 80.741) that provides the resulting value of 4.17 mm (Fig 3). This result confirms that, in many cases, even with a deviated midline, one could still have a beautiful smile and it could also explain the divergence among the results found by the various authors in their respective studies. 

The almost perfect negative linear correlation (r = -0.9963) between the means and the deviations, demonstrated that the higher the deviation, the lower was the score assessed by the evaluators, and vice-versa. The coefficient of determination (r^2^ = 0.9926) indicates that 99.26% of the variation of the mean scores ​​assigned to the photographs can be explained by the amount of deviation. The evaluators were able to perceive the increase of the deviation despite the randomization of photographs.

This study is clinically important to the extent that it provides scientific data that makes it easier for professionals to better understand the patient’s esthetic expectations and desires. Thus, it helps to outline the treatment plan and define which procedures should be performed during the final stage of orthodontic treatment. One last caveat is necessary: professionals should be aware that in some cases dental midline correction can prove a daunting task, which can involve complicated mechanic and result in increased complexity and duration of orthodontic treatment.

## CONCLUSIONS

1) The laypersons were able to perceive the upper dental midline deviations of 1 mm and above when the adjacent structures of the smiles were viewed; and of 2 mm and above when only the lips were viewed.

2) Visualization of structures adjacent to the smile, such as lips, chin and nose demonstrated influence on the perception of upper dental midline deviation.
